# Efficacy and Tolerability of Tenofovir Disoproxil Fumarate Based Regimen as Compared to Zidovudine Based Regimens: A Systematic Review and Meta-Analysis

**DOI:** 10.1155/2017/5792925

**Published:** 2017-05-30

**Authors:** Tegene Legese Dadi, Adane Teshome Kefale, Teshale Ayele Mega, Muktar Sano Kedir, Habtamu Acho Addo, Tessema Tsehay Biru

**Affiliations:** ^1^College of Health Sciences, Mizan-Tepi University, Mizan Teferi, Ethiopia; ^2^Institute of Health Sciences, Jimma University, Jimma, Ethiopia

## Abstract

**Background:**

Although tenofovir (TDF)/emtricitabine (FTC)/efavirenz (EFV) and zidovudine (ZDV)/lamivudine (3TC)/efavirenz (EFV) are used as preferred first line regimen, their head-to-head comparison in terms of their efficacy and tolerability was limited. This review aimed to synthesize the best available evidence on the comparative efficacy and tolerability of the two regimens.

**Methods:**

Seven sites and databases in addition to Google search until August 20, 2016, were searched. Only randomized clinical trials conducted on adult population were included in this study. Our primary outcome was viral load suppression while secondary outcomes were death and tolerability. Undetectable viral load is defined as <50 Human Immunodeficiency Virus (HIV) ribonucleic acid (RNA) copies/ml. Joanna Briggs institute meta-analysis of statistics assessment and review instrument (JBI-MAStARI) and critical appraisal and data extraction tool were applied for critical assessment and data extraction, respectively. We performed a random effect meta-analysis to pool the relative risk (RR) for viral load suppression (<50 HIV RNA copies/ml and <400 HIV RNA copies/ml), tolerability, and death.

**Result:**

Data was extracted from four articles, which included a total of 2381 participants. We found superior viral load suppression among tenofovir (TDF) arm compared to zidovudine (ZDV) arm. Tenofovir arm achieves viral load <50 HIV RNA copies/ml (RR = 1.12, 95% confidence interval (CI) [1.04, 1.21], *I*^2^ = 0%) higher than zidovudine arm. Similarly TDF arm is superior in viral load suppression to <400 HIV RNA copies/ml (RR = 1.19, 95% CI [1.11, 1.27], *I*^2^ = 0%). Moreover, TDF based regimens were more likely to be tolerated than ZDV based regimens (4 trials, 2381 participants (RR = 1.06, 95% CI [1.02, 1.10], *I*^2^ = 51%)). However, forest plot of death shows that it was not significant (RR = 0.91, 95% CI [0.51, 1.62]).

**Conclusion:**

The use of TDF/FTC/EFV as first line regimen for naïve HIV-1 infected adult patient showed superior viral load suppression and tolerability as compared to ZDV/3TC/EFV. In order to compare the death outcome of both ZDV/3TC/EFV and TDF/FTC/EFV further research is needed.

## 1. Background

Since its advent in 1986, Human Immunodeficiency Virus (HIV), as a causative agent for Acquired immunodeficiency syndrome (AIDS), became one of the greatest threats to public health [1, 2]. In 2015, there were 2.1 million new infections worldwide and 36.7 million people living with HIV/AIDS (PLWHA). Globally, from 33.3 million PLWHA in 2010 [3, 4] 32.6 million of them were from low-and middle-income countries [5]. Fortunately, with the introduction of antiretroviral therapy (ART) [6], it is now possible to control HIV, and discovery of combination ART had further revolutionized the HIV care and treatment [2].

With the continued scale-up of access to ART, at the end of 2015, the global ART coverage reached 46%. It was increased from 24% in 2010 to 54%, in eastern and southern Africa [5]. A rising number of countries have committed to achieving the 90-90-90 treatment target by 2020, as set by the World Health Organization (WHO) in 2015 using the currently available combination ART regimens [7].

Because of rapidly emerging scientific understanding of HIV treatment, care, and the dynamic scale-up efforts in resource limited settings, the WHO guidelines are updated routinely every few years. So the 2013 WHO ART guideline brought TDF based regimens in clinical practice, together with ZDV based regimens [8, 9]. Therefore, ZDV/3TC/NVP (zidovudine plus lamivudine plus nevirapine) or EFV (efavirenz) and TDF/3TC (FTC)/EFV (tenofovir plus lamivudine or emtricitabine plus efavirenz or NVP (nevirapine)) are continued to be the most important components of ART regimens in resource constrained settings [1].

Even though the current treatments are largely manageable, issues of tolerability and efficacy continue to be a concern, especially when combination therapy has been taken [10]. Literatures reported that TDF + 3TC + NVP was associated with higher hazard of mortality and virologic failure as compared to AZT + 3TC + NVP [11]. In another study, TDF based regimens are protective [12], despite it predisposing patients into safety issues associated with kidney and bone [13]. In other literatures, there were no statistically significant differences in all-cause mortality [14] and risk of HIV-1 disease progression or death in both arms [15]; contrary to these facts, TDF based regimens have been found to have a high genetic barrier to resistance, better effectiveness, favorable toxicity profile, and demonstrated regimen durability [8, 9, 16, 17].

The fractions of evidence on which ART guideline is revised need to be organized for further synthesis. Systematic reviews serve as the basis for compiling and assessing the evidence upon which these recommendations are updated. The two previously published systematic reviews comparing the tolerability and efficacy of TDF versus ZDV based regimens were unable to show the true picture of the two currently recommended first line combination ART regimens. In addition a review by Omeje and Okwundu 2012 [14] included only a single clinical trial for its conclusion. Besides this a review by Spaulding et al. 2010 [9] included two trials. It does not show the true picture of tolerability and efficacy of TDF versus ZDV based regimens since it lacks head-to-head comparison of the drugs. This limitation forced the authors to recommend further reviews that involved head-to head comparison for further best evidence synthesis. Therefore, in this meta-analysis, we compared the two regimens in a head-to-head fashion by using available randomized clinical trials.

## 2. Methods

### 2.1. Search Strategy and Selection of Reviews

After having a common search strategy, all six authors independently and systematically searched to identify articles on tolerability and treatment outcomes of TDF and ZDV based regimens published until August 20, 2016. All authors searched each database on the same day to be consistent. Search terms included HIV, tenofovir, and zidovudine with their MeSH terms.

In order to identify articles published in the English language that compare TDF and ZDV based regimen, we searched the PubMed, EMBASE, Scopus, Google Scholar, Mednar and Natural Standard alternative medicine, and Clinical Trials.Gov. We did the initial search for articles on July 11–18, 2016, and updated the results on August 20, 2016.

Articles were eligible for inclusion if they contain only randomized clinical trials which included a head-to-head comparison of tenofovir based regimen with zidovudine based regimens among treatment naïve adult (age ≥ 18 years) HIV-1 infected patients. From both regimens the nucleoside reverse transcriptase inhibitors (NRTI) backbone other than comparison of interest can be either lamivudine or emtricitabine. Lamivudine and emtricitabine are comparable in efficacy and safety. There were no restrictions on publication date and country of focus.

We excluded articles that contained only nevirapine based regimens from nonnucleoside reverse transcriptase inhibitors (NNRTI) or compared nevirapine based regimens with efavirenz based or protease inhibitor based regimens to minimize risk of confounding.

Besides above-mentioned inclusion criteria, papers that meet the inclusion criteria are critically appraised by two independent reviewers for methodological validity using standardized critical appraisal instruments from the Joanna Briggs institute meta-analysis of statistical assessment and review instrument (JBI-MAStARI) (Appendix  I in Supplementary Material available online at https://doi.org/10.1155/2017/5792925). Any discrepancies that come up between the commentators will be decided through discussion or with a third reviewer.

Undetectable viral load is defined as <50 Human Immunodeficiency Virus (HIV) ribonucleic acid (RNA) copies/ml.

### 2.2. Primary Study Identification and Data Extraction

We extracted the evidence from original articles if they assessed at least one of the following outcomes: viral load suppression, death, and tolerability. Viral load suppression to either below <400 HIV RNA copies/ml or to undetectable levels (<50 HIV RNA copies/ml) was considered as primary outcome while secondary outcomes were death and tolerability. We extracted outcome using the similar data extraction tool of JBI-MAStARI (Appendix  II). All results were taken out by two independent reviewers to avoid extraction error.

### 2.3. Data Analysis

We entered data into review manager 5.3 for analysis and we did a random effect and fixed-effect meta-analysis to pool the relative risk (RR) of the outcomes. We performed a random effect meta-analysis to pool the relative risk (RR) for viral load suppression (<50 HIV RNA copies/ml and <400 HIV RNA copies/ml), tolerability, and death.

Forest plot containing RR, 95% confidence intervals (CI), *P* value, effect size, and heterogeneity (*I*^2^) were constructed. *P* values less than 0.05 were considered statistically significant.

## 3. Result

**Figure 1:**
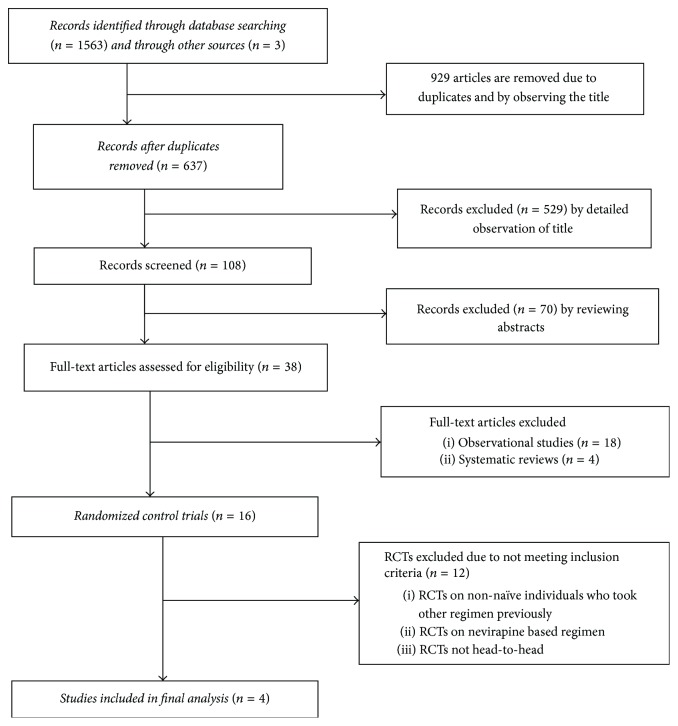
Flow chart of study selection.

A total of 1566 articles were identified from the different databases. After removal of duplicates and removal of articles by title and abstract thirty-eight full text papers were identified for eligibility. Twenty-two of the papers were rejected due to not meeting the inclusion criteria, and the rest 16 of them were examined for relevance. Of the 16 articles, we excluded 12 articles by critical appraisal. Four articles were included in the final meta-analysis (Figure 1).

**Figure 2:**
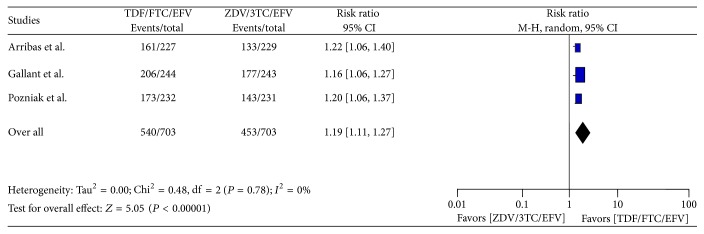
Forest plot of viral load suppression to less than 400 HIV RNA copies/ml in the TDF arm as compared to ZDV arm in ART naïve HIV-1 infected patients.

From three articles, data of 1406 patients (703 from TDF/FTC/EFV and 703 from ZDV/3TC/EFV) with 1 : 1 in the treatment and control group was extracted for determining the viral load suppression to less than 400 HIV RNA copies per milliliter. The numbers of patients who achieved this viral load suppression in the two arms were found to be 540 (76.81%) and 453 (64.44%) in treatment and control groups, respectively. Test of overall effect showed 1.19 times likelihood of achieving viral load suppression to less than 400 HIV RNA copies/ml in TDF arm compared to ZDV arm, (RR = 1.19, 95% CI [1.11, 1.27]) without heterogeneity: Tau^2^ = 0.00, Chi^2^ = 0.48, df = 2 (*P* = 0.78); *I*^2^ = 0%, with test for overall effect of *Z* = 5.05  (*P* < 0.00001) (Figure 2).

**Figure 3:**
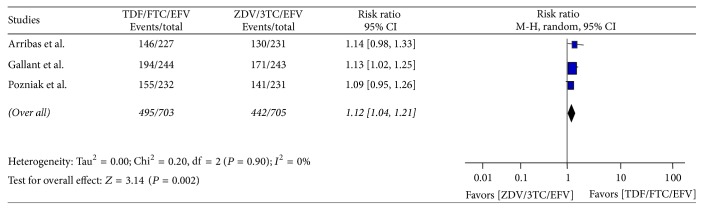
Forest plot of viral load suppression to less than 50 HIV RNA copies/ml in TDF arm as compared to ZDV arm in ART naïve HIV-1 infected patients.

Similarly data of 1408 patients (703 from TDF/FTC/EFV and 705 from ZDV/3TC/EFV) in 1 : 1 in both groups was obtained for assessing the viral load suppression to undetectable levels (HIV RNA < 50 copies/ml). The numbers of patients who achieved this viral load suppression in the two arms were found to be 495 (70.41%) and 442 (62.69%) in treatment and control groups, respectively. Test of overall effect showed 1.12 times more likelihood of achieving undetectable viral load suppression of TDF arm compared to ZDV arm, (RR = 1.12, 95% CI [1.04, 1.21]) without heterogeneity: Tau^2^ = 0.00, Chi^2^ = 0.20, df = 2 (*P* = 0.90); *I*^2^ = 0%, with test for overall effect of *Z* = 3.14(*P* < 0.002) (Figure 3).

The observed homogeneity (*I*^2^ = 0%) among the studies may empower synthesizing the best evidence for concluding more likelihood of viral load suppression to any extent (whether to undetectable levels (<50 HIV RNA copies/ml) or to <400 HIV RNA copies/ml) among patient treated with TDF/FTC/EFV compared to those treated ZDV/3TC/EFV regimen. Therefore TDF/FTC/EFV has statistically superior efficacy profile in preventing virologic failure than ZDV/3TC/EFV combination ART regimen.

**Figure 4:**
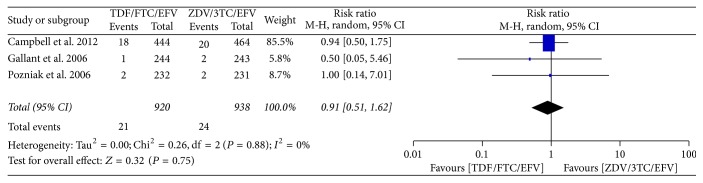
Forest plot of mortality effect of TDF arm as compared to ZDV arm in ART naïve HIV-1 infected patients.

Data of 1858 patients (920 from TDF/FTC/EFV (treatment) and 938 from ZDV/3TC/EFV (control)) with nearly 1 : 1 in the treatment and control group was obtained for assessing the mortality outcome. The number of patients who died in the two groups was calculated to be 21 (2.3%) and 24 (2.6%) in treatment and control groups, respectively. The cumulative RR between the two arms did not confer any statistical significance, (RR = 0.91, 95% CI [0.51, 1.62]) without heterogeneity: Tau^2^ = 0.00, Chi^2^ = 0.26, df = 2 (*P* = 0.88); *I*^2^ = 0%, and the test for overall effect showed that *Z* = 0.32 (*P* = 0.75) (Figure 4).

The observed homogeneity (*I*^2^ = 0%) among the studies may empower synthesizing the best evidence for concluding the absence of risk difference in mortality among patient treated with TDF/FTC/EFV and ZDV/3TC/EFV regimens. Therefore the two arms have statistically equivalent efficacy profile in preventing death; however, clinically significant mortality differences may exist.

**Figure 5:**
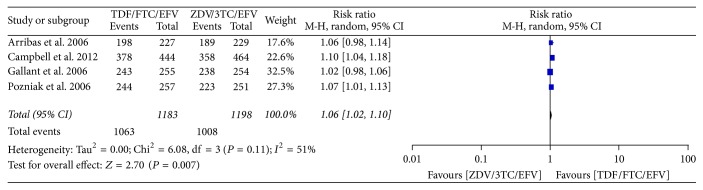
Forest plot of tolerability of TDF arm as compared to ZDV arm in ART naïve HIV-1 infected patients.

We also compared the tolerability of the two arms using a data of 2,381 patients (1183 from TDF/FTC/EFV and 1198 from ZDV/3TC/EFV) with nearly 1 : 1 in the treatment and control group. The numbers of patients who tolerated the regimens until the end of the study were calculated to be 1063 (89.86%) and 1008 (84.14%) in TDF/FTC/EFV and ZDV/3TC/EFV arms, respectively. Test of overall effect revealed 1.06 times likelihood of tolerating TDF arm as compared to ZDV arm, (RR = 1.06, 95% CI [1.02, 1.10]), Chi^2^ = 6.08, df = 3 (*P* = 0.11); *I*^2^ = 51%, *Z* = 2.70 (*P* < 0.007) (Figure 5). The observed homogeneity (*I*^2^ = 51%) among the studies may empower synthesizing the best evidence for concluding more tolerability among patient treated with TDF/FTC/EFV than ZDV/3TC/EFV combination ART regimen (Figure 5).

## 4. Discussion

We included three clinical trials that enrolled a total of 1408 participants, four clinical trials that enrolled a total of 2381 participants, and three clinical trials that enrolled a total of 1858 participants for determining efficacy, tolerability, and mortality, respectively.

In this meta-analysis, we found superiority viral load suppression among the TDF/FTC/EFV arm as compared to ZDV/3TC/EFV arm in achieving undetectable viral load (<50 HIV RNA copies/ml) (RR = 1.12, 95% CI [1.04, 1.21], *P* = 0.002), and viral load suppression to <400 HIV RNA copies/ml (RR = 1.19, 95% CI [1.11, 1.27], *P* < 0.00001). This finding contradicted the pervious meta-analysis and systematic review [9, 14]. The possible reason for this difference might be due to small sample size and heterogeneity of the studies included the previous studies. Beside this, the previous meta-analysis and systematic review did not conduct head-to-head comparison of the two arms, which might increase the risk of confounding on their conclusion. Moreover, TDF/FTC/EFV arm showed better tolerability as compared to ZDV/3TC/EFV arm (RR = 1.06, 95% CI [1.02, 1.10], *Z* = 2.70, *P* < 0.007, *I*^2^ = 51%). This finding is similar to the previous studies [8, 9, 14]. These findings reveal an increasing evidence on the superiority of TDF/FTC/EFV in terms of efficacy and tolerability as first line regimen for naïve HIV-1 infected adult patients and support the inclination toward the preference of TDF/FTC/EFV as initial ART regimen over ZDV/3TC/EFV, due to its cost effectiveness and lower pill burden, besides superior efficacy and better tolerability [18–21].

On the other hand, the cumulative risk ratio of death between the two arms did not confer any statistical significance (RR = 0.91, 95% CI [0.51, 1.62]); this result was similar to the previous systematic review [14]. This might be due to death being a rare outcome; thus the precision to detect RR is low. The ideal method to compare the death outcome between the two arms is to include case control studies.

The strength of this study includes head-to-head comparison of the two regimens, homogeneity of included studies, use of only randomized clinical trials for evidence synthesis, and determining efficacy in terms of viral load suppression, which is considered as the gold standard of efficacy measurement. The limitation of this was due to the fact that pooling of the results measured on different follow-up periods, 48, 96, and 144 weeks, on the same population might affect the result.

## 5. Conclusion

The use of TDF/FTC/EFV as first line regimen for naïve HIV-1 infected adult patient showed superior viral load suppression and tolerability as compared to ZDV/3TC/EFV. In order to compare the death outcome of both ZDV/3TC/EFV and TDF/FTC/EFV further research is needed.

## Supplementary Material

The Joanna Briggs institute meta-analysis of statistical assessment and review instrument (JBI-MAStARI) is developed by Adelaide University, Australia. The critical appraisal contains all necessary components to appraise clinical trial articles.

## Abbreviations

AIDS: Acquired immunodeficiency syndrome
ART: Antiretroviral therapy
CI: Confidence intervals
EFV: Efavirenz
HIV: Human Immunodeficiency Virus
NVP: Nevirapine
PLWHA: People living with HIV AIDS
RR: Relative risk
TDF: Tenofovir disoproxil fumarate
TDF/3TC (FTC)/EFV: Tenofovir disoproxil fumarate plus Lamivudine or Emtricitabine plus Efavirenz
TDF/3TC (FTC)/NVP: Tenofovir disoproxil fumarate plus lamivudine or emtricitabine plus nevirapine
WHO: World Health Organization
ZDV: Zidovudine
ZDV/3TC/NVP: Zidovudine plus lamivudine plus nevirapine
ZDV/3TC/EFV: Zidovudine plus lamivudine plus efavirenz.
