# Supplementary material for: Efficacy and Tolerability of Tenofovir Disoproxil Fumarate Based Regimen as Compared to Zidovudine Based Regimens: A Systematic Review and Meta-Analysis
Source: AIDS Res Treat. 2017 May 30;2017:5792925. doi: 10.1155/2017/5792925 (PMC5468550; doi:10.1155/2017/5792925)
Supplement: Supplementary file 1 — The Joanna Briggs institute meta-analysis of statistical assessment and review instrument (JBI-MAStARI) is developed by Adelaide University, Australia. The critical appraisal contains all necessary components to appraise clinical trial articles. [file 5792925.f1.docx]

**Appendix I**

**Appraisal instrument**

**
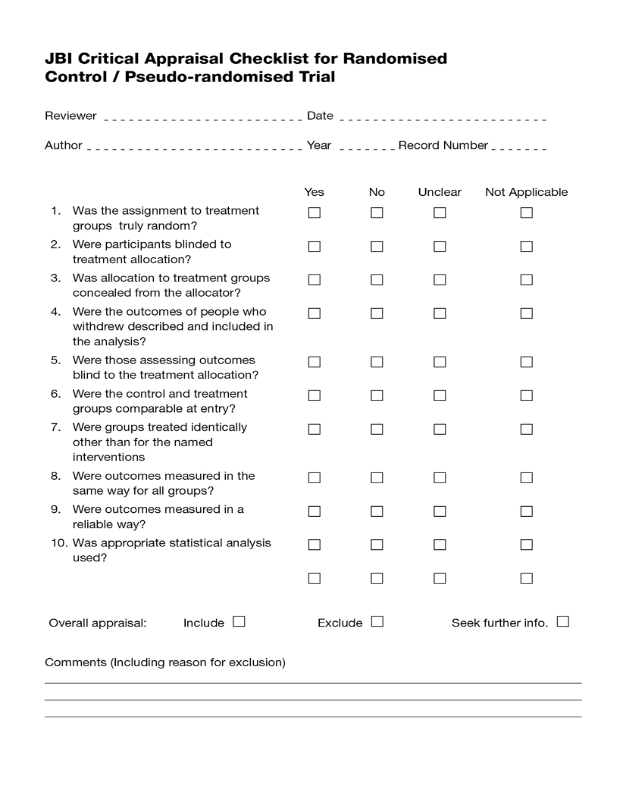
**

**Appendix II**

**Data extraction format**


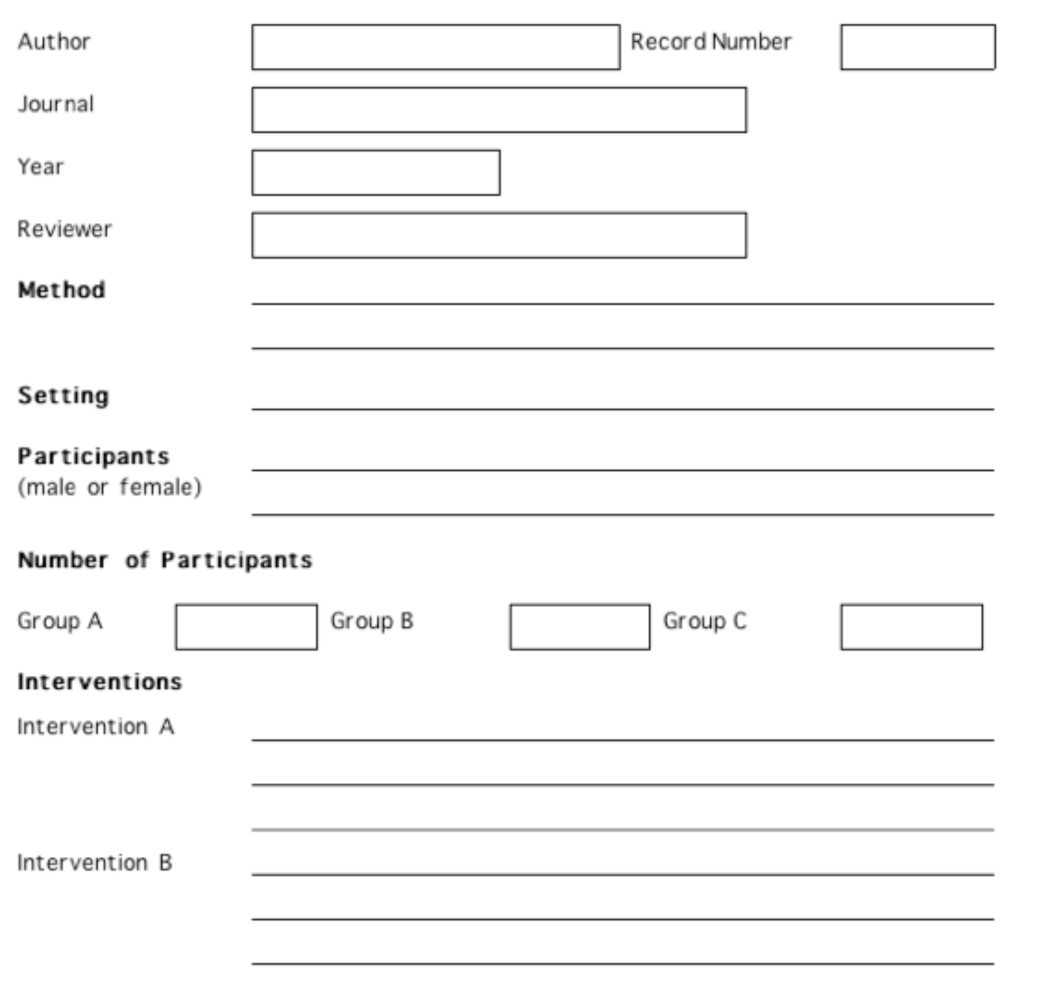

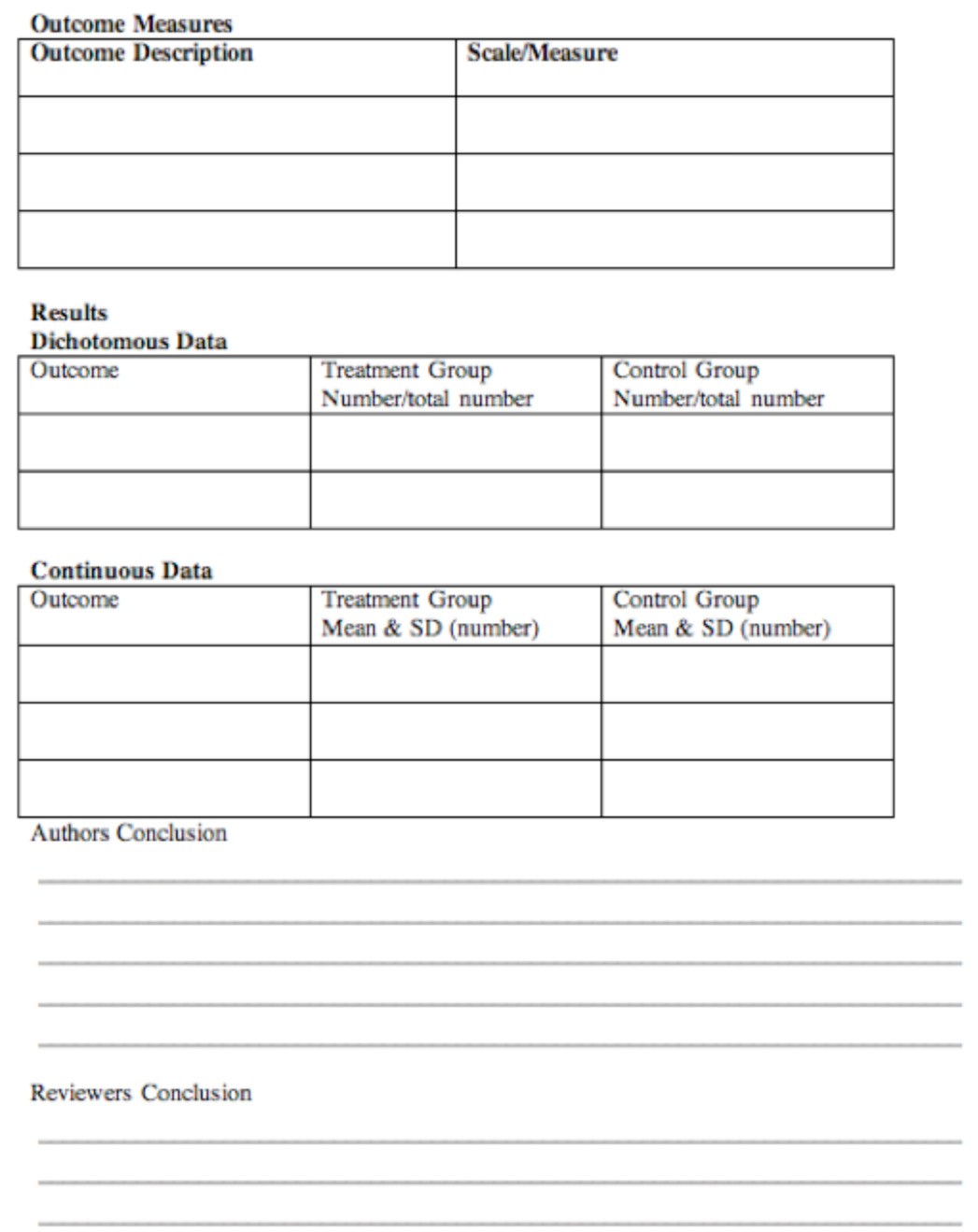


**Appendix III**

**Search terms on PubMed**

Search (((((((((((((tenofovir) OR (R) -9- (2-phosphonylmethoxypropyl) adenine) OR 9- (2-Phosphonomethoxypropyl) adenine) OR 9- (2-Phosphonylmethoxypropyl) adenine) OR 9- (2-Phosphonylmethoxypropyl) adenine, isomer) OR 9- (2-Phosphonylmethoxypropyl) adenine, (R) isomer t357098) OR 9- (2-Phosphonylmethoxypropyl) adenine, (S) isomer) OR 9-PMPA (tenofovir)) OR tenofovir disoproxil) OR tenofovir disoproxil fumarate) OR viread)) AND (((((((((zidovudine) OR 3'-Azido-2',3'-Dideoxythymidine) OR 3'-Azido-3'-deoxythymidine) OR Azidothymidine) OR AZT (Antiviral)) OR AZT Antiviral) OR BW A509U) OR BWA-509U) OR Retrovir)) AND ((((((((((((((((((HIV) OR Acquired Immune Deficiency Syndrome Virus) OR Acquired Immunodeficiency Syndrome Virus) OR AIDS Virus) OR HTLV-III) OR Human Immunodeficiency Virus) OR Human Immunodeficiency Viruses) OR Human T Cell Lymphotropic Virus Type III) OR Human T Lymphotropic Virus Type III) OR Human T-Cell Leukemia Virus Type III) OR Human T-Cell Lymphotropic Virus Type III) OR Human T-Lymphotropic Virus Type III) OR Immunodeficiency Virus, Human) OR Immunodeficiency Viruses, Human) OR LAV-HTLV-III) OR Lymphadenopathy-Associated Virus) OR Virus, Human Immunodeficiency) OR Viruses, Human Immunodeficiency) Filters: Clinical Study; Clinical Trial; Comparative Study; Meta-Analysis; Observational Study; Randomized Controlled Trial; Review; Systematic Reviews; Humans; English; AIDS; Adult: 19+ years; Adolescent: 13-18 years
